# Everybody's Page

**Published:** 1888-08-25

**Authors:** 


### Everybody's Page.

It is said that between 3,000 and 4,000 lbs. of caffeine are annually made in Germany from damaged tea which has been rejected by the British custom houses.

According to Mr. Keldyche, who has just published the results of a series of experiments on the air drawn from hospital wards, air which has been saturated with eucalyptol will no longer permit the propagation of colonies of bacilli.

Coca is now being grown in India, and although the leaves obtained from the plants differ in chemical constitution from those of the South American variety, their physiological activity is unimpaired. This promises a new source of income for our Indian Empire.

An enterprising firm of French chemists publish as a testimonial a letter from M. Pasteur, stating that the "peptone" which forms their specialty is admirably adapted for the cultivation of microbes, and giving an order for some to be used for this purpose. On the whole, we don't think this is the sort of recommendation which will induce simple people to buy M. Chapoteaut's peptone for their own consumption.

Antipyrine is now employed in Paris to an almost incredible extent. The average French d ctor prescribes it for all the ills that flesh is heir to; it has become as necessary an article in every lady's boudoir as her perfume-bottle; the very children have had so much of it now that they cry out for it as they might for their meals. This amateur drugging is one of the most reprehensible of modern follies.

Dr. Von Mosetig-Monbrof, speaking at the Vienna Medical Congress, showed himself to be possessed of great originality in one thing—his bold and unique treatment of the English language. He quoted the following as Lister's statement of his "fundamental satze": 1. "Let the wound to be alone;" 2. "Let the wound to be protected;" 3. "Let to the wound her free discharge." This interesting specimen of "English as she is spoke" by Sir Joseph Lister will doubtless surprise many people; not least, we should imagine, Sir Joseph Lister himself.

The legislative assembly of the State of New York passed a law, on June 21st, forbidding any druggist to make up more than once any prescription containing more than one-fourth of a grain of opium, or one-twentieth of a grain of morphine without a special order from the physician who originally prescribed the dose. This severe restriction is rendered necessary by the increasing tendency of many patients who have once had morphia administered medicinally to continue to take it as a stimulant. This is one of the most insidious forms of intoxication, and is indulged in by many who would regard the idea of taking too much alcohol with horror.

Milk is heavier than water, and so heavy milk is supposed to be good; but as cream is lighter than milk, a milk which had been skimmed would show better on the lactometer than would a genuine milk. And yet these instruments are used and implicitly believed in as milk-testers by many otherwise well-informed people, and it is said, and probably truly enough, that at one time certain French officials, whose duty included that of examining milk, were all armed with these lactometers. Thus equipped, they perambulated the streets, and whenever they thought proper they demanded a sample of milk from any dealer. Into this they plunged their lactometer, and if the milk did not come up to the mark it was forthwith poured into the gutter. In this way, no doubt, many a gallon of good milk was lost.

Attention has lately been called to an acid extracted from *Gymnema sylvestris*, a climbing plant from India. This acid deprives the tongue for the time being of the power of distinguishing sweet from bitter substances. It does not interfere with the taste of saline substances.

Typhoid fever has, upon the whole, diminished both relatively and absolutely. In 1886 the percentage of deaths from typhoid fever to deaths from all causes was 2·15; in the year of greatest prevalence, within a period of twenty years —1872—the percentage of deaths from typhoid to deaths from all causes was 4·86, and the death-rate per 1,000 living was 11·1. The diminution in the death-rate from this disease has been especially marked in cities with new water supplies and good systems of sewers.

In a recent communication to the Academy of Medicine on alcoholism and criminality, M. Maramhas stated that in examining the history of three thousand criminals undergoing sentences of various lengths, he found that of the vagabonds and beggars there were 79 per cent. who were confirmed drunkards; of asassins and incendiaries from 50 to 57 per cent.; of thieves and swindlers 71 per cent., while of those convicted of violence to the person there were 88 per cent., and 79 per cent. guilty of violence to property.

The best time of the day for sea-bathing is about two or three hours after eating, and preferably in the forenoon. It may be borne in mind that the beach and the waves themselves are generally cleaner during the ebb tide than during the flood; and also that it is desirable that the air, as well as the sea, should be warm when one is bathing. The first bath of the season should be a brief one, lasting no longer than is necessary to wet the body from head to foot. In bathing, as in other things, custom hardens; and at the end of his holiday the bather may remain in the water with impunity for a length of time that would have been highly dangerous for him a few weeks earlier.

There is a disease called trichinosis, in which the muscles become filled with little living organisms—small worm-like bodies, a condition which is comparatively rare in this country, but common in certain parts of Germany, and is produced by eating badly-cooked pork. The parasite infests the pig, and in some parts of America a large proportion of the pigs are affected by it. The living organism in the pork can, fortunately, be killed by a sufficient amount of heat. The pork should not only be roasted—for when roasted a crust or hard coating is apt to be formed on the outside of the joint, and the organisms in the inner parts of the mass to be unkilled—but should be first thoroughly boiled and then roasted. The preliminary boiling does not, as one might suppose, spoil the dish; in fact, to many people's taste, it improves it.

Without any special ventilation at all, but leaving the room door ajar by a single inch, or at most by two inches, and having the chimney vent open, then the ventilation will proceed in a somewhat satisfactory manner, still more so if the window can be opened a little. If the lower sash be raised, and a slip of wood be inserted on which the window can be closed down, it will leave a ventilating space between the lower and upper sashes, which will be found most effective and beneficial as a ventilator without much down-draught, as the entrance of the outside air will be directed upwards as it passes into the room. An ordinary window sash, 3 feet wide, if raised in this way by a single inch, will give 36 square inches of ventilating surface, and if the air blowing at 2 miles an hour, it will pass 2,700 cubic feet per hour.

